# miR-34a regulates mesangial cell proliferation via the PDGFR-β/Ras-MAPK signaling pathway

**DOI:** 10.1007/s00018-014-1599-y

**Published:** 2014-03-18

**Authors:** Dapeng Chen, Ying Li, Yan Mei, Wenjia Geng, Jurong Yang, Quan Hong, Zhe Feng, Guangyan Cai, Hanyu Zhu, Suozhu Shi, Xue-Yuan Bai, Xiangmei Chen

**Affiliations:** 1 State Key Laboratory of Kidney Diseases (2011DAV00088), Department of Nephrology, Chinese PLA Institute of Nephrology, National Clinical Research Center for Kidney Disease (2013BAI09B05), Chinese PLA General Hospital, 28 Fuxing Road, Beijing, 100853 People’s Republic of China; 2 Medical College, NanKai University, Tianjin, People’s Republic of China; 3 Department of Nephrology, Third Hospital, Hebei Medical University, Shijiazhuang, People’s Republic of China; 4 Department of Nephrology, Daping Hospital, Research Institute of Surgery, Third Military Medical University, Chongqing, People’s Republic of China

**Keywords:** Mesangial proliferative glomerulonephritis (MsPGN), miR-34a, PDGFR-β, Ras/MAPK signaling pathway

## Abstract

The main pathological characteristic of glomerulonephritis is diffuse mesangial cell proliferation. MiR-34a is associated with the proliferation of various organs and cancer cells. However, the role of miR-34a in renal proliferation diseases is not clear. Therefore, this study aimed to elucidate the mechanism of miR-34a in the regulation of renal mesangial cell proliferation. The miR-34a expression level at different time points in an anti-Thy1 mesangial proliferative nephritis rat model was determined by qRT-PCR. The cell proliferation rate and cell cycle changes were measured in the in vitro cultured rat mesangial cells (RMCs). Our results suggested that miR-34a expression was negatively correlated with the degree of cell proliferation in the anti-Thy1 nephritis model. MiR-34a could extend the G0/G1 phase and block cell proliferation in RMCs. Dual-luciferase assay results showed that there were binding sites of miR-34a at 3′-UTR of platelet-derived growth factor receptor-β (PDGFR-β). MiR-34a can inhibit PDGFR-β protein expression at a post-transcriptional level, suppress Ras/MAPK signaling pathways, and down-regulate expression of cell cycle proteins at the G0/G1 phase, such as cyclin D1, CDK4/CDK6. In addition, miR-34a may also inhibit RMC proliferation by directly targeting cyclin E and CDK2. MiR-34a inhibits exogenous stimuli-induced proliferation of mesangial cells. Expression levels of phospho-PDGFR-β and phospho-MEK1 (an important downstream molecule in PDGFR-β-induced signaling pathway) were significantly increased in the anti-Thy-1 nephritis rat model. These results suggest that miR-34a may regulate RMC proliferation by directly inhibiting expressions of PDGFR-β, MEK1, and cell cycle proteins, cyclin E and CDK2.

## Introduction

Mesangial proliferative glomerulonephritis (MsPGN) can be divided into IgA nephropathy and non-IgA nephropathy, which show diffuse hyperplasia of glomerular mesangial cells (GMCs) and different degrees of extracellular matrix (ECM) accumulation [1, 2]. Inhibition of mesangial cell proliferation is an important treatment strategy in proliferating glomerular diseases [3].

Anti-Thy1 nephritis is a rat model of human mesangial proliferative glomerulonephritis, involving a combination of Thy1 antibody and Thy1 antigen in mesangial cells. Macromolecular immune complexes are formed in situ and deposited in the mesangial area, leading to mesangial cell proliferation and inflammation [4, 5]. These inflammatory mediators in turn stimulate activation, shrinkage, and proliferation of mesangial cells; release a variety of inflammatory mediators and ECM components [6, 7]; and aggravate inflammation, all of which result in glomerular damages, including damage to the molecular barrier and charge barrier of the filtration membrane, and proteinuria [8].

MicroRNAs (miRNAs) are 21- to 25-nucleotide (nt)-long, single-stranded RNA molecules that serve as post-transcriptional regulators of gene expression in eukaryotes and that do not code for any protein [9–11]. miRNAs can regulate the expression of thousands of proteins by degrading target mRNA or inhibiting translation as a result of complementary matching between miRNAs and specific sites in target genes [12]. miRNAs are widely involved in a series of biological functions and are closely associated with human diseases, including kidney diseases [13].

miR-34 was first found in the worm *Caenorhabditis elegans* in 2001 [14]. In recent years, it has been shown that miR-34a is involved in tumor proliferation of neuroblastoma [15, 16], colon cancer [17], uveal melanoma [18], brain tumors [19], and cervical cancer [20] through regulation of different target genes. However, the role of miR-34a in mesangial proliferative glomerulonephritis is unclear. We thus aimed to investigate the role of miR-34a in renal proliferation diseases.

## Materials and methods

### Anti-Thy1 nephritis animal model

Male Wistar rats (Beijing Vital River Laboratory Animal Technology Co., Ltd., Beijing, China) weighing between 200 and 220 g were randomly allocated to the control and anti-Thy1.1 groups. Anti-Thy1.1 nephritis was induced by a single intravenous injection of a monoclonal anti-Thy1 antibody (2.5 mg/kg) produced by OX-7 cells. Controls were injected with an identical volume of normal saline. Anti-Thy1-treated animals were killed on days 3, 5, 7, 10, and 14 post-injection (*n* = 3 per time point), and the control animals were killed on day 0 (*n* = 3). All rats were provided a diet of standard laboratory chow and free access to water. The renal tissues were examined by routine periodic acid-Schiff staining.

### Immunohistochemical analysis and evaluation

The renal tissues were fixed in formalin and embedded with paraffin. The histologic paraffin sections were cut to 3- to 4-μm thickness and mounted on poly-l-lysine-coated slides. Endogenous peroxidase was blocked with 3 % hydrogen peroxide. The sections were heated in a microwave oven for 10 min in sodium citrate buffer (pH 6.0), incubated with 1.5 % normal goat serum for 20 min, and incubated overnight with 1:100 diluted primary antibody (Ki-67). For a negative control, the sections were incubated with PBS. After removal of unbound primary antibody, the sections were incubated with a biotinylated secondary antibody for 60 min at room temperature. The sections were rinsed and incubated with avidin-biotinylated horseradish peroxidase (Vectastain Elite ABC Kit; Vector Laboratories, USA) for 60 min. Incubation with 3,3-diaminobenzidine tetrahydrochloride was performed for 10 min as a substrate chromogen solution to produce a brown color. Finally, the sections were counterstained with hematoxylin. Twenty glomeruli per section at each time point were evaluated under high-power light microscopy (×400) in a blinded fashion. The Ki67 labeling index was calculated as the number of Ki-67-positive cells to total glomerular cells.

### Cell culture and transfection

Rat mesangial cells (RMCs) were purchased from the American Type Culture Collection (ATCC). Cells were cultured in RMPI 1640 medium (Gibco, USA) containing 10 % fetal bovine serum (FBS; Hyclone, Canada), 100 U/ml penicillin, and 100 μg/ml streptomycin (Gibco, USA) at 37 °C in a humidified incubator with an atmosphere containing 5 % CO_2_. Trypsin (0.25 %) was used for cell passages. RMCs (~50 % confluence) were transfected with miR-34a mimics (Dharmacon, Thermo Scientific, USA; cat. no. C-300551-07), miRNA control (Dharmacon cat. no. CN-001000-01), siPDGFR-β or siCon (Table 1) using jetPRIME (Polyplus-transfection, USA) for 48 h, followed by determination of protein and RNA levels.

**Table 1:** siRNA sequence

Target gene		5′ → 3′
siPDGFR-β	Sense	GAC GCU GCA UGA GAA GAA ATT
	Antisense	UUU CUU CUC AUG CAG CGU CTT
siCon	Sense	UUC UCC GAA CGU GUC ACG UTT
	Antisense	ACG UGA CAC UGG CGG AGA ATT

### Quantitative reverse-transcriptase polymerase chain reaction (qRT-PCR)

TaqMan miRNA assays were performed to quantify mature miR-34 levels. Briefly, cDNA was synthesized using Taqman RT reagents (Applied Biosystems, USA), followed by TaqMan-based quantitative PCR using specific mirVana qRT-PCR primer sets. The primary transcript levels of PDGFR-β, cyclin D1, cyclin E, CDK2, CDK4, CDK6, and p27^kip1^ (Table 2) were determined with the SYBR Green Real-time qPCR Master Mix (Toyobo, Japan). The qRT-PCR analyses were conducted using an Applied Biosystems ABI Prism 7300 Sequence Detection System, and qRT-PCR reactions were performed using the following parameters: 95 °C for 10 min, followed by 40 cycles at 95 °C for 15 s and 60 °C for 1 min. The relative RNA levels were calculated using the ΔΔ*C*_t_ method and normalized to U6 snRNA control for rat miR-34a or to GAPDH for RMC mRNAs. Sequence-specific primers for the indicated genes were designed using the Primer Premier software, version 5.0 (PREMIER Biosoft International, USA).

**Table 2:** Real-time qPCR primers for genes

Target gene		5′ → 3′	Product
PDGFR-β	Sense	ACC ATG CGG GCC TTC CAT GC	152 bp
	Antisense	TTT GGC TGA GGC ATG CCC CG	
Cyclin D1	Sense	CTG CAG CTT CTG GGG GCC AC	119 bp
	Antisense	AGC AAC TCC TCG GGG CGG AT	
Cyclin E	Sense	ATT CAG CGT GCG TGG ACC CC	119 bp
	Antisense	GGA GGC TCT GGG CGG TCT GA	
CDK2	Sense	TTC CCC AAG TGG GCT CGG CA	128 bp
	Antisense	GCC AGG GCT GCT TTG GCT GA	
CDK4	Sense	TCC GAG CCC TGC AGC ACT CTT A	152 bp
	Antisense	AGG CTG CCA CTT CAG CAA GGT T	
CDK6	Sense	ACT TCG GCC TTG CCC GCA TC	79 bp
	Antisense	TCC GGG GCT CGG TAC CAC AG	
p27	Sense	CAC CCC AAG CCT TCC GCC TG	108 bp
	Antisense	GCG CTG GCT CGC TTC TTC CA	
GAPDH	Sense	GGC ATG GAC TGT GGT CAT GAG	87 bp
	Antisense	TGC ACC ACC AAC TGC TTA GC	

### Cell proliferation assay

As a measure of cell proliferation, the number of viable cells was detected using the Cell Counting Kit-8 (Beyotime, China). RMCs were seeded in 96-well plates at a density of 2,000/well, synchronized by incubation in FBS(−) medium for 12 h, and then transfected with miR-34a mimics or a miRNA control. At 24, 48, and 72 h post-transfection, the number of viable cells was measured by recording the optical density at 450 nm and generating growth curves. Experiments were performed in triplicate, and sextuplet wells were used.

### Cell cycle analysis

RMCs were seeded in six-well plates at a density of 1.0 × 10^5^/well, synchronized by incubation in FBS(−) medium for 12 h, and then transfected with miR-34a mimics or a miRNA control for 48 h. The cells were washed twice with PBS, suspended in 75 % ethanol, and fixed by incubation in 75 % ethanol at 4 °C overnight. Fixed cells were collected by centrifugation, washed with PBS, treated with RNase (50 μg/ml; Sigma, USA), and stained with propidium iodide (50 μg/ml; Sigma, USA). A FACS flow cytometer (BD Co., USA) was used to determine cellular DNA contents. The percentage of cells in G0/G1, S, and G2/M phases were determined using the Cell FIT Cell Cycle Analysis software (version 2.01.2; BD).

### Western-blot analysis

Proteins were extracted from RMCs using RIPA lysis buffer (50 mM Tris–HCl, pH 7.5, 150 mM NaCl, 0.5 % deoxycholate, 1 % Nonidet P-40, 0.1 % SDS, 1 mM PMSF, and protease cocktail at 1 μg/ml). Protein concentrations were determined using a BCA kit (Pierce, USA). Protein samples (60 μg per lane) were separated by 10 or 12 % SDS-PAGE electrophoresis and transferred to NC membranes. After staining with Ponceau S, the membranes were incubated overnight in 5 % non-fat milk at 4 °C, followed by incubation with primary antibodies against PDGFR-β, p-PDGFR-β, and p27^kip1^ (Cell Signaling Technology, USA); cyclin D1, cyclin E, CDK2, CDK4, CDK6, ERK1/2, and p-ERK1/2 (Abcam, USA); MEK1 and p-MEK1 (Abcam, USA); Raf1, p-Raf1 and K-ras(Abnova, USA); or β-actin (Sigma, USA). Immunoreactive bands were visualized using ECL reagent (Santa Cruz Biotechnology), according to the manufacturer’s instructions, and exposure to X-ray film. Protein band intensities were quantified using Quantity One software (Bio-Rad, USA).

### Plasmids

PDGFR-β, cyclin E, and CDK2 cDNAs were amplified from RMCs by PCR. The 1521-bp fragment of the PDGFR-β 3′-UTR, the 426-bp fragment of the cyclin E 3′-UTR, or the 999-bp fragment of the CDK2 3′-UTR (Table 3), all containing the miR-34a targeting sequence, was cloned into the psiCHECK dual-luciferase reporter plasmid (Promega, USA; cat. no. C8021) to produce psiCHECK-WT-PDGFR-β, psiCHECK-WT-cyclin E, or psiCHECK-WT-CDK2, respectively, using PCR. Sequence-specific primers for the indicated genes were designed using the Primer Premier software, version 5.0. The sequences of all constructs were verified.

**Table 3:** PCR primers for 3′-UTR fragments

Target gene		5′ → 3′	Product
PDGFR-β	Sense	CTG CCC AGG ACC TCT GGC TG	2548 bp
	Antisense	CAG AAG GGC CTCC CAG GAG GG	
CDK2	Sense	TCT CTG GGC CAG CCG GTT CT	999 bp
	Antisense	ACA ACA CGG GGT GGG AGA AGG A	
Cyclin E	Sense	TCC TCC TCC GAG TGG GGT CCT	426 bp
	Antisense	ACA GCA ACC CTG CGA CAC CC	

### Dual-luciferase reporter assay

For reporter assays, RMCs were cultured to approximately 80 % confluence in a 24-well plate and then co-transfected with a dual-luciferase reporter plasmid (psiCHECK-PDGFR-β, or psiCHECK-cyclin E, or psiCHECK-CDK2) and miR-34a mimics for 48 h. The activities of firefly and Renilla luciferases were determined using the Dual-Luciferase Reporter Assay System (Promega cat. no. E1910), and firefly luciferase activity was normalized to that of Renilla luciferase.

### Statistical analyses

Measurement data are expressed as mean ± SEM, unless otherwise stated. Statistical analyses were performed using the SPSS 15.0 software package (SPSS, Inc., USA). Comparison among groups was conducted with ANOVA. Statistical differences were evaluated with analysis of variance. A *p* value <0.05 denoted a statistically significant difference.

## Results

### Pathological changes in rat model of anti-Thy1 mesangial proliferative glomerulonephritis

**Fig. 1:**
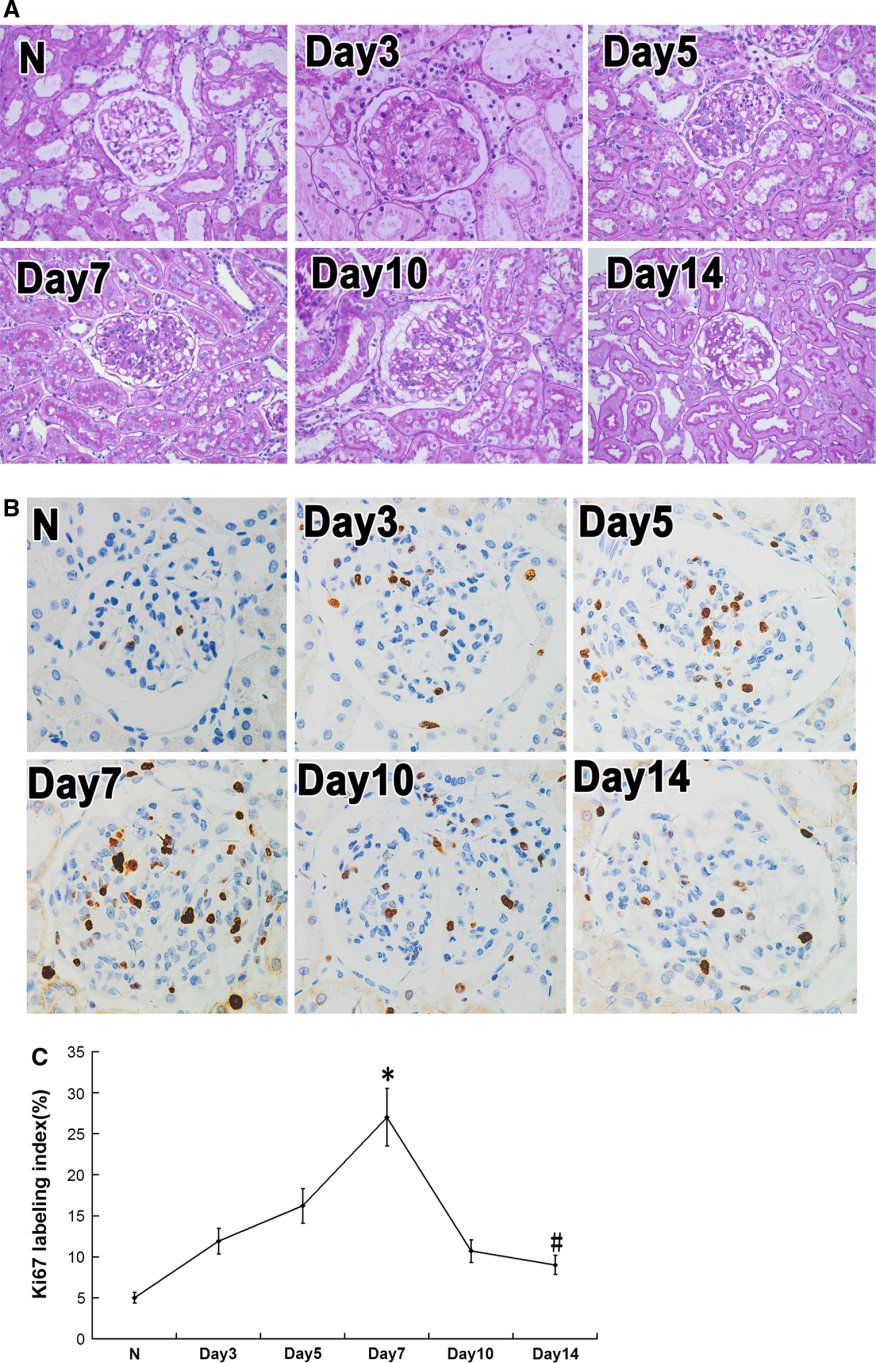
Pathological changes and expression of Ki-67 in anti-Thy1 nephritis model rats. **a** Periodic acid-Schiff stain (original magnification ×400). **b** Expression of Ki-67 in anti-Thy1 nephritis model rats. Expression of Ki-67 was detected with immunohistochemistry analysis. **c** Ki-67 labeling index was calculated by Ki-67-positive cells to total glomerular cells. N: normal group rats; day 3, 5, 7, 14, and 28: anti-Thy1 nephritis rats at day 3, 5, 7, 14, 28, separately. *p < 0.05 vs. N (normal group rats). ^#^ *p* < 0.05 vs. Day7 group, *n* = 6

We injected Thy1 antibody into Wistar rats to create an anti-Thy1 mesangial proliferative glomerulonephritis rat model. Following injection of anti-Thy1 antibodies, partial complement-dependent mesangiolysis appeared on day 3; mesangial cell proliferation and ECM accumulation occurred on day 5 and peaked on day 7. On day 10, recovery from glomerular injury began to decrease, and ECM accumulation attenuated on day 14 (Fig. 1a). We also detected the expression changes of cell proliferation marker Ki-67 by immunohistochemistry (Fig. 1b, c). We found higher levels of Ki-67 at every time point during anti-Thy1 nephritis compared with the control, which suggests that the cell cycle remained active from days 3 to 10. Ki-67 increased on days 3 and 5, peaked on day 7, and decreased from days 10 to 14. This suggests that cell cycle activity increased from days 3 to 7 and subsequently gradually decreased from days 10 to 14 (*p* < 0.05)(Fig. 1b, c).

### MiR-34a expression in the kidney tissues of anti-Thy1 nephritis rat model

**Fig. 2:**
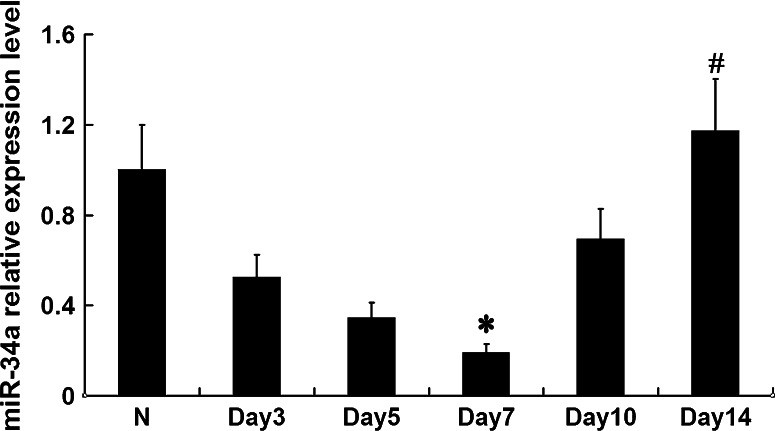
MiR-34a expression levels were detected with qRT-PCR in anti-Thy1 model rats. *p < 0.05 vs. N (normal group), *n* = 6; ^#^ *p* < 0.05 vs. day 7 group, *n* = 6

MiR-34a expression was detected in the kidney tissues of the anti-Thy1 nephritis rat model at various time points (days 3, 5, 7, 10, and 14) by real-time qPCR. Compared with the normal group, miR-34a expression gradually decreased as pathological changes progressed from day 3 (*p* < 0.05). Expression levels and pathologic outcomes became normal on day 14 (*p* < 0.05) (Fig. 2).

### Effect of miR-34a on mesangial cell proliferation rate and cell cycle

**Fig. 3:**
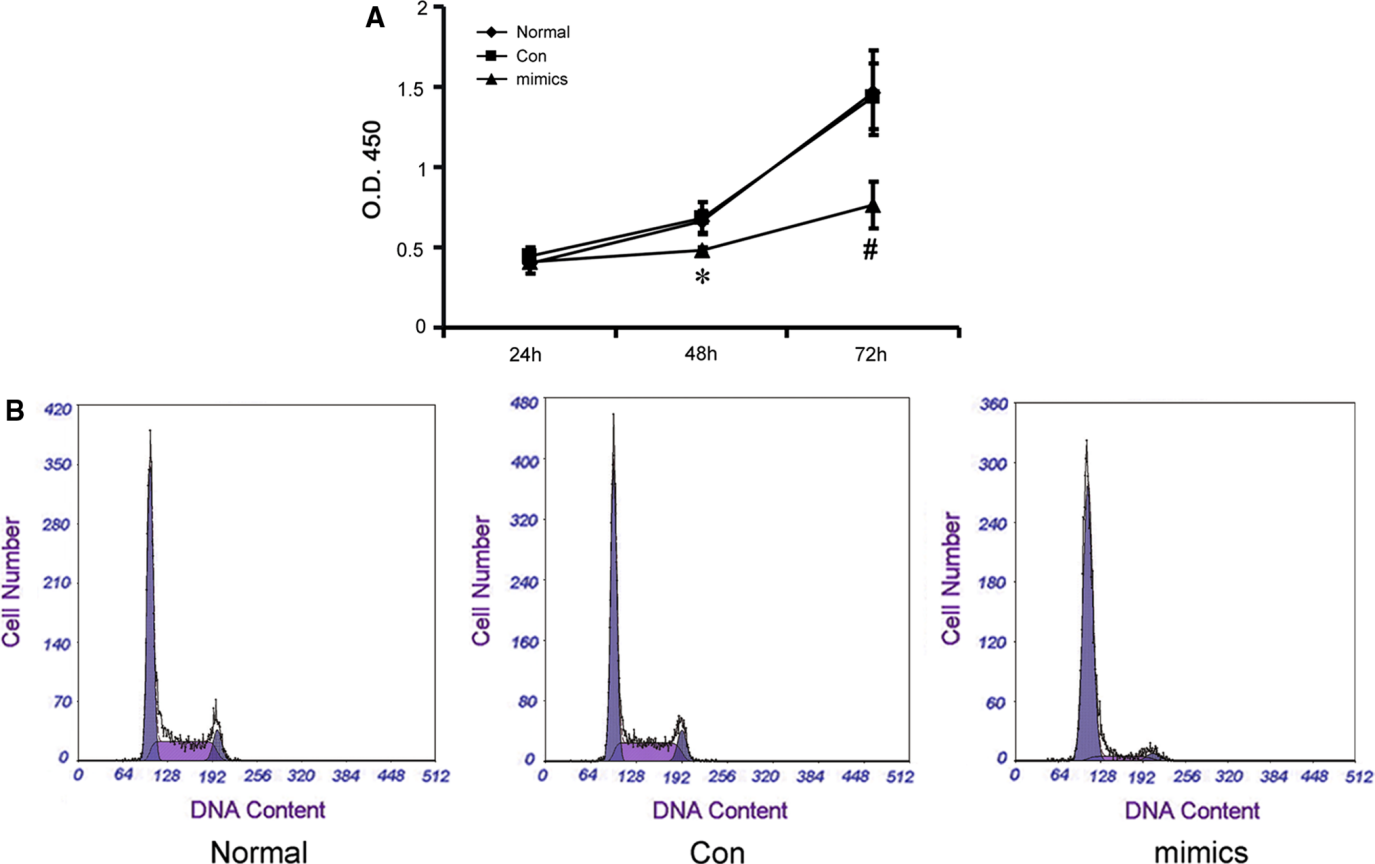
Changes of proliferation activity and cell cycle in the RMCs transfected with miR-34a mimics. **a** Proliferation activity of RMCs was detected by Cell Counting Kit-8 after transfection with miR-34a mimics or miRNA-negative control. *p < 0.05 vs. normal (normal group) and Con (miRNA-negative control group) at 48 h, *n* = 3; ^#^ *p* < 0.05 vs. normal group and Con group at 72 h, *n* = 3. Mimics: miR-34a mimics. **b** Analysis results of cell cycle by flow cytometry method (FCM) in the RMCs transfected with microRNA-34a mimics or miRNA-negative control

RMCs were transfected with miR-34a mimics (mimics group) or a miRNA-negative control (Con group), and the proliferation rate of miR-34a mimic-transfected cells was determined using the Cell Counting Kit-8 (Fig. 3a). Proliferation of RMCs was reduced at 24 h (*p* > 0.05), significantly reduced at 48 h (*p* < 0.05), and more obvious at 72 h (*p* < 0.01) in miR-34a mimics-transfected group, compared with the normal group and the miRNA-negative control group, indicating that miR-34a mimics influence RMC proliferation. Cellular DNA content was further assessed by flow cytometry. The results showed that miR-34a mimics markedly extended the G0/G1 phase and reduced the G2/M phase (Fig. 3b; Table 4), compared with the normal group and the miRNA-negative control group (*p* < 0.05).

**Table 4:** The percentage of mesangial cells in G0/G1, S, and G2/M phases

	G0–G1 (%)	S (%)	G2-M (%)
Normal	63.86 ± 2.31	23.16 ± 1.62	12.98 ± 1.57
Con	61.07 ± 1.74	24.14 ± 2.26	14.79 ± 1.30
Mimics	75.81 ± 2.19*	16.68 ± 1.24*	7.52 ± 0.79*

*Normal* normal group, *Con* miRNA-negative control group, *Mimics* miR-34a mimics group

* *p* < 0.05 vs. normal and Con, *n* = 3

### Influence of miR-34a on possible target gene, PDGFR-β

**Fig. 4:**
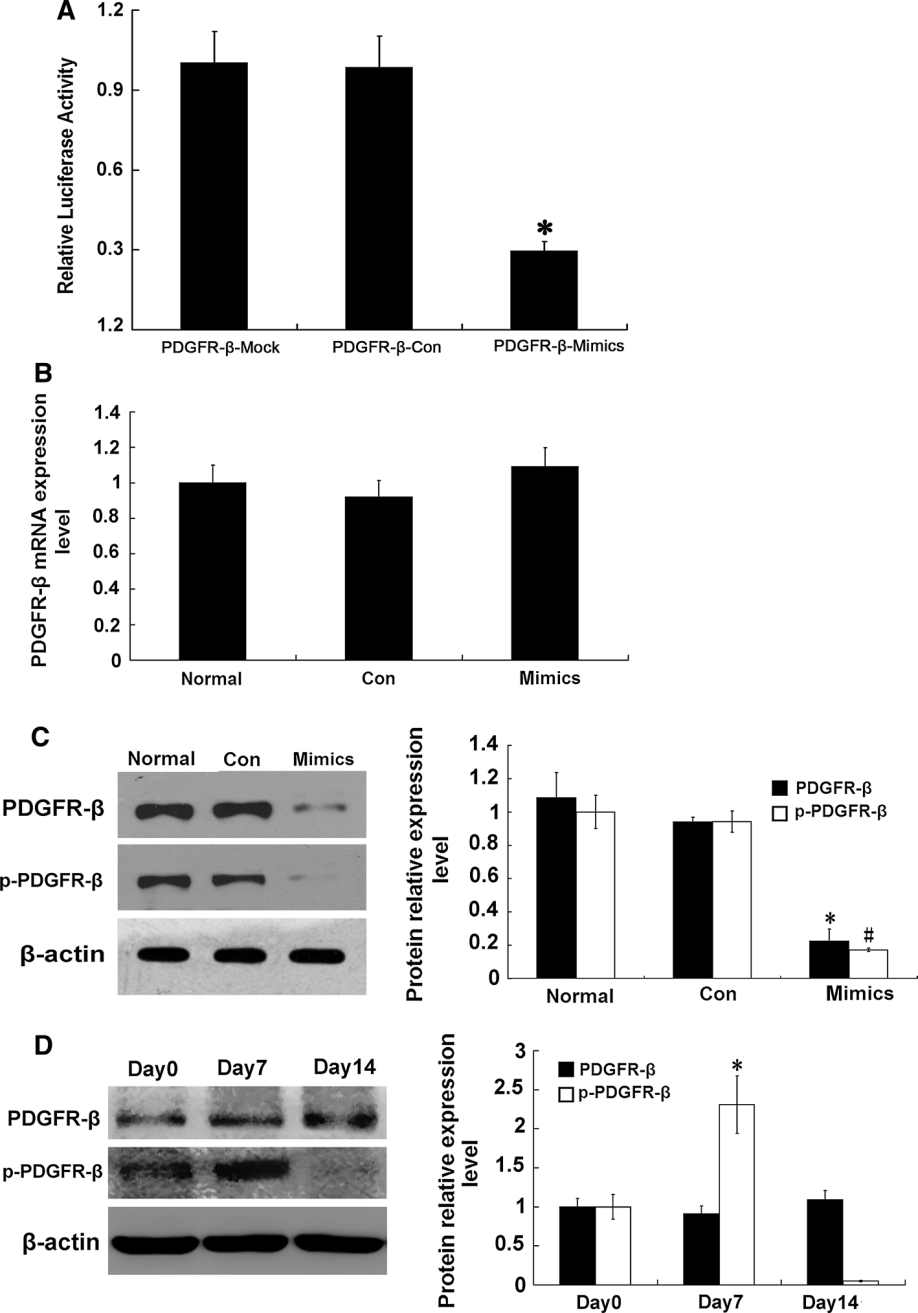
Effect of miR-34a on expression of PDGFR-β. **a** Effect of miR-34a on expression of PDGFR-β by using Dual-Luciferase Reporter plasmids. The 3′-UTR of PDGFR-β was inserted into 3′ end of luciferase coding sequence in psiCHECK vector and recombinant plasmid was transfected into RMCs with miR-34a mimics or miRNA-negative control. Firefly luciferase activity was normalized to Renilla luciferase activity. *p < 0.01 vs. PDGFR-β-Mock (psiCHECK vector) and PDGFR-β-Con (psiCHECK-PDGFR-β+miRNA-negative control). PDGFR-β-Mimics: psiCHECK-PDGFR-β+miR-34a mimics. **b** Expression level of PDGFR-β mRNA was detected with real-time qPCR after transfection with miR-34a mimics or miRNA-negative control. Normal: normal group; Con: miRNA-negative control group; Mimics: miR-34a mimics. **c** Protein expression levels of PDGFR-β and phospho-PDGFR-β were detected with Western blot after transfection with miR-34a mimics or miRNA-negative control. Data are means of three separate experiments ± SD. *^, #^ *p* < 0.05 vs. normal group and Con group, *n* = 3. Normal: normal group; Con: miRNA-negative control group; Mimics: miR-34a mimics. **d** Protein expression levels of PDGFR-β and phospho-PDGFR-β were detected with Western blot on day 0, day 7, and day 14 in anti-Thy1 model rats. Data are means of three separate experiments ± SD. *p < 0.05 vs. day 0 and day 14, *n* = 3

By using online biology software miRGen (http://www.diana.pcbi.upenn.edu/miRGen.html), we hypothesized that PDGFR-β may be a target gene of miR-34a (Table 5). To confirm whether miR-34a interacts with the 3′-UTR of PDGFR-β and regulates target gene expression at a post-transcriptional level, we cloned the 3′-UTR fragment of PDGFR-β into the dual-luciferase reporter vector psiCHECK to construct a psiCHECK-PDGFR-β plasmid, which was co-transfected into RMCs with miR-34a mimics or miRNA-negative control. MiR-34a mimics significantly down-regulated firefly luciferase activity in psiCHECK-PDGFR-β-transfected cells (*p* < 0.05) (Fig. 4a). To further confirm the direct action of miR-34a, levels of PDGFR-β mRNA and protein were determined in the miR-34a mimics-transfected cells. There was no significant difference between miR-34a mimic-transfected cells and control cells in terms of PDGFR-β mRNA expression level (*p* > 0.05) (Fig. 4b). Western blot showed that protein expressions of PDGFR-β and phospho-PDGFR-β were significantly decreased in the miR-34a mimic-transfected cells compared with the control cells (*p* < 0.05) (Fig. 4c). These results indicate that PDGFR-β may be a target gene of miR-34a. Moreover, miR-34a likely inhibits PDGFR-β primarily through post-transcriptional regulation. At the same time, we detected protein expressions of PDGFR-β and phospho-PDGFR-β (activated PDGFR-β) in the anti-Thy-1 nephritis rat model. Compared with day 0, on day 7 (the most obvious pathological change), phospho-PDGFR-β was significantly increased (*p* < 0.05) (Fig. 4d), indicating that PDGFR-β plays a key regulative role in proliferation development in the anti-Thy-1 nephritis.

**Table 5:**
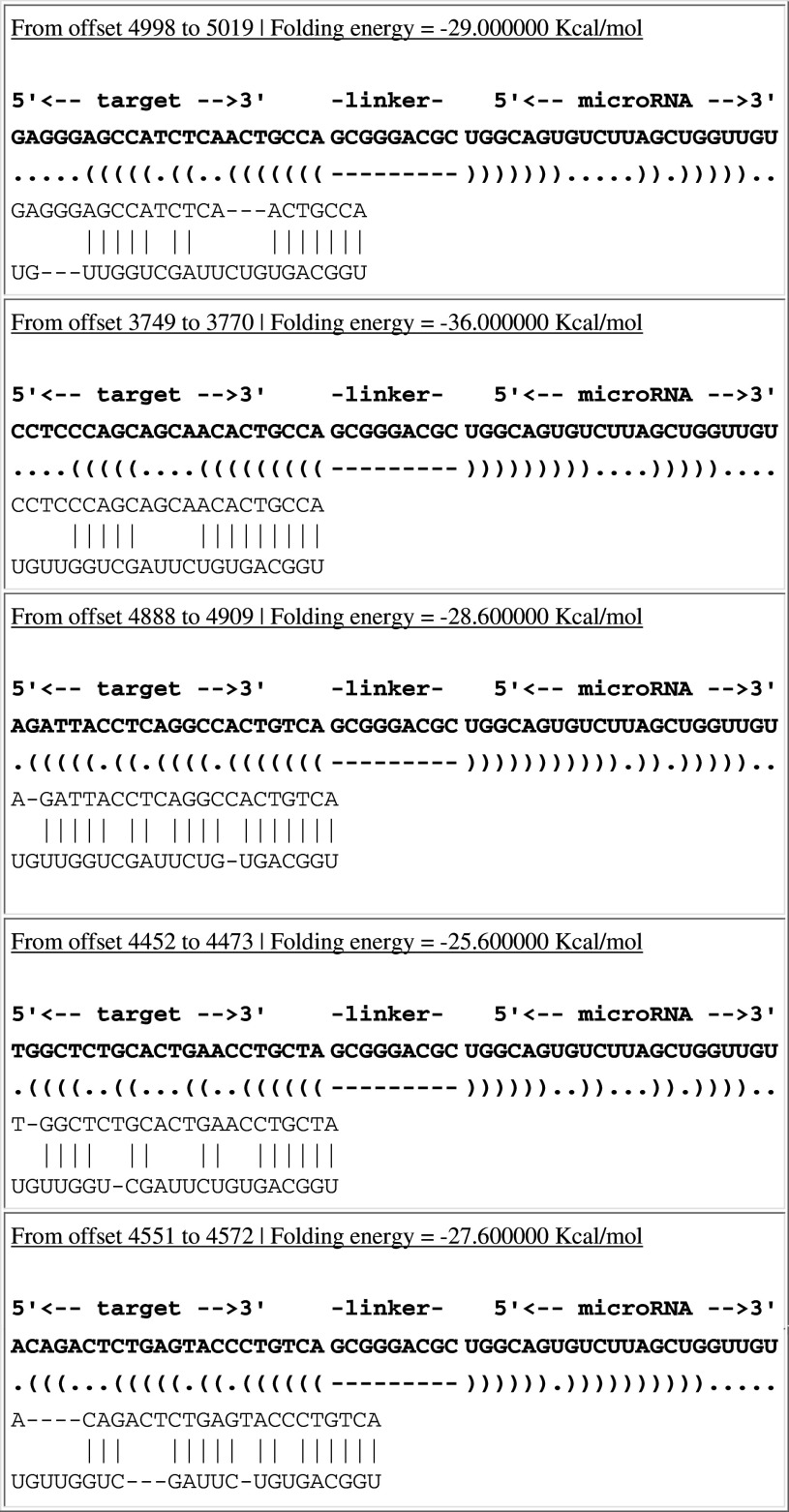
The combined sequence of miR-34a target PDGFR-β in 3′UTR

### Effects of miR-34a on expressions of cell cycle-related mRNAs and proteins

**Fig. 5:**
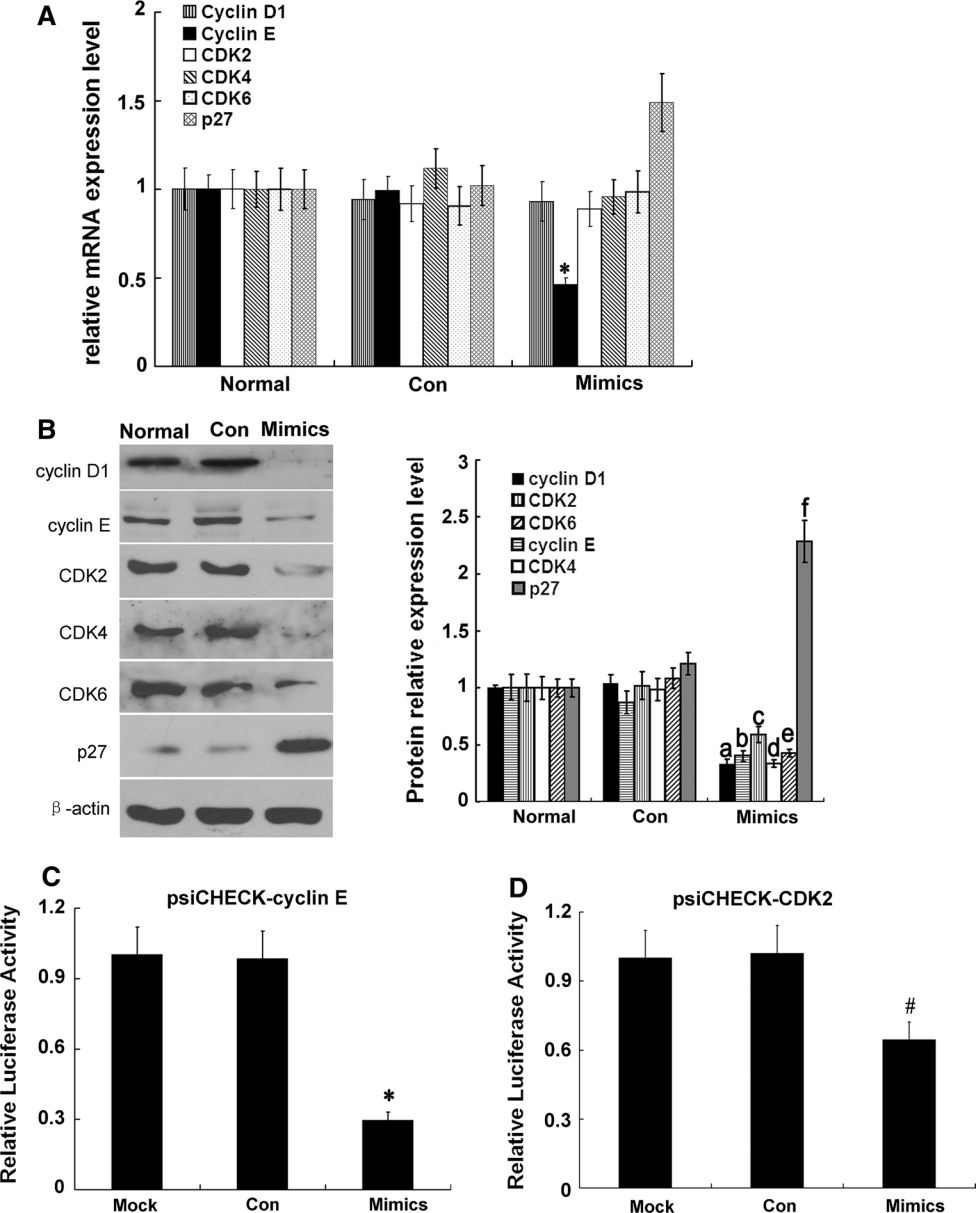
Expression of G0/G1 phase cell cycle-related proteins in RMCs after transfection with miR-34a mimics. **a** MiRNA expression levels of cyclin D1, cyclin E, CDK2, CDK4, CDk6, p27^kip1^ were detected with qRT-PCR. *p < 0.05 vs. normal (normal group) and Con (miRNA-negative control group). **b** Proteins expression levels of cyclin D1, cyclin E, CDK2, CDK4, CDk6, p27^kip1^ were detected with Western blot. Data are means of three separate experiments ± SD. ^a,b,c,d,e,f^ *p* < 0.05 vs. normal group and Con group, *n* = 3. Mimics represented miR-34a mimics. **c** Effect of miR-34a on expression of cyclin E. Data are means of three separate experiments ± SD. *p < 0.01 vs. Mock (psiCHECK-vector) and Con (psiCHECK-cyclin E + negative control). Mimics: psiCHECK-cyclin E + miR-34a mimics. **d** Effect of miR-34a on expression of CDK2. Data are means of three separate experiments ± SD. ^#^ *p* < 0.01 vs. Mock (psiCHECK-vector) and Con (psiCHECK-CDK2 + negative control). Mimics: psiCHECK-CDK2 + miR-34a mimics

Compared with the normal group and Con group, cyclin E mRNA level was significantly decreased (*p* < 0.05), and that of P27^kip1^ was significantly increased (*p* < 0.05); however, there were no changes in mRNA levels of cyclin D1, CDK2, CDK4, or CDK6 in the miR-34a-transfected cells (*p* > 0.05) (Fig. 5a). Levels of cell cycle proteins (cyclins D1 and E), CDK proteins (CDK2, CDK4, and CDK6), and CKI (p27^kip1^) were also determined. Levels of cyclin D1, cyclin E, CDK2, CDK4, and CDK6 were significantly reduced (*p* < 0.05), while that of p27^kip1^ was significantly increased (*p* < 0.05) in the miR-34a-transfected cells, compared with the normal group and Con group (Fig. 5b). Two cell-cycle complexes, cyclin D1/CDK4/CDK6 and cyclin E/CDK2, mainly play a role during the mid G0/G1 stage. Previous studies showed that cyclin D1 [21], CDK4 [9], and CDK6 [21, 22] are target genes of miR-34a. Therefore, we hypothesized that cyclin E and CDK2 may be target genes of miR-34a. We cloned the 3′-UTRs of cyclin E and CDK2 into psiCHECK-2 to construct two plasmids, psiCHECK-cyclin E and psiCHECK-CDK2. Both miR-34a mimics and the two plasmids were co-transfected into cells. MiR-34a significantly down-regulated firefly luciferase activity in psiCHECK-2-cyclin E-transfected cells (Fig. 5c) and psiCHECK-2-CDK2-transfected RMC cells (Fig. 5d), suggesting cyclin E and CDK2 to be target genes of miR-34a.

### Influence of miR-34a on the molecules in the Ras/MAPK signaling pathway

**Fig. 6:**
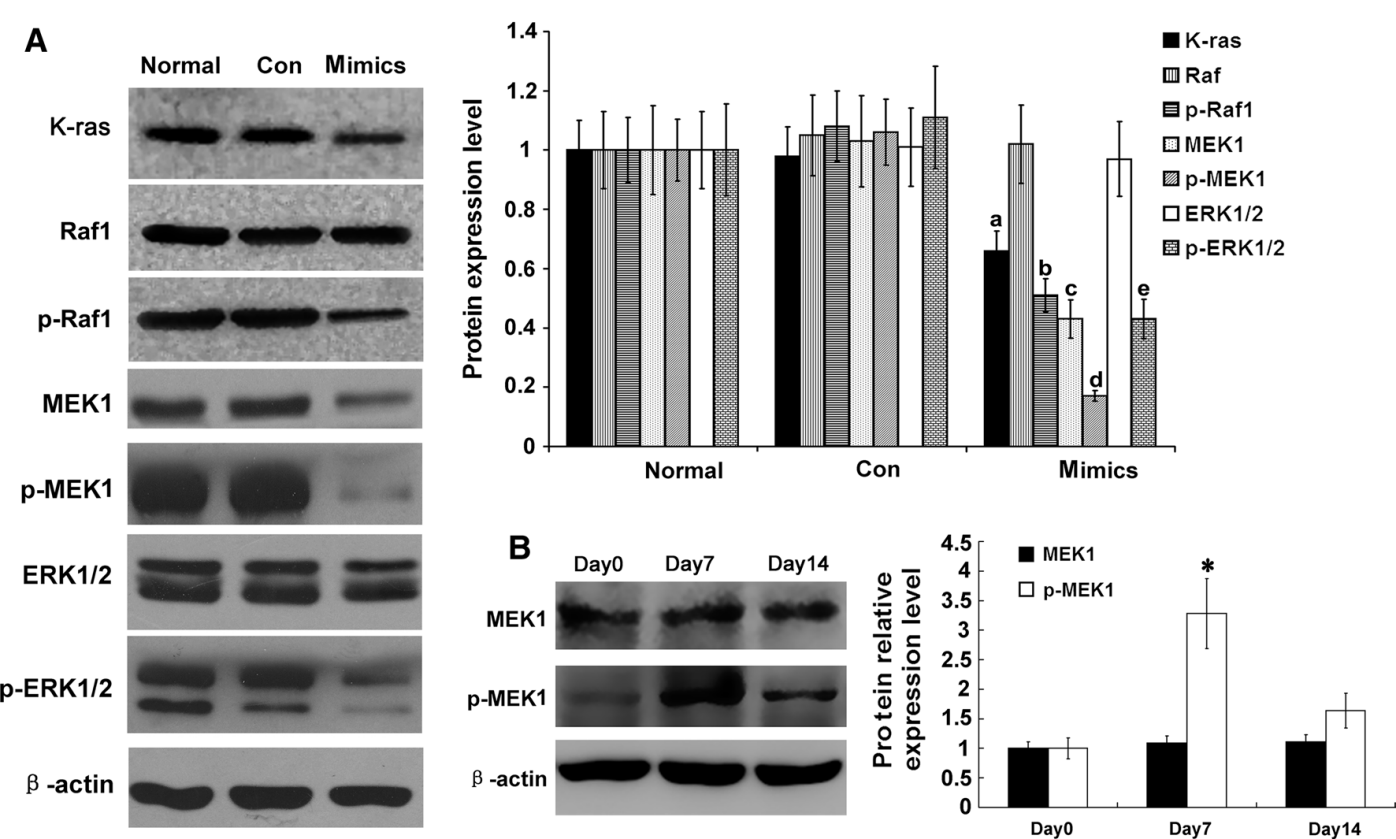
Expression levels of Ras/MAPK signaling pathway-related proteins were detected with Western blot in RMCs after transfection with miR-34a mimics. **a** Protein expression levels of K-ras, Raf-1, p-Raf-1, MEK1, p-MEK1, ERK, p-ERK were detected with Western blot. Data are means of three separate experiments ± SD. ^a,b,c,d^ *p* < 0.05 vs. normal group and Con group (miRNA-negative control group), *n* = 3. Mimics: miR-34a mimics. Renilla luciferase activity was normalized to firefly luciferase activity. **b** Protein expression levels of MEK1 and p-MEK1 were detected with Western blot. Data are means of three separate experiments ± SD. *p < 0.01 vs. day 0 and day 14, *n* = 3

RMCs were transfected with miR-34a mimics or a miR-negative control, and expression levels of two key targets in the Ras/MAPK signaling pathways were determined. The total MEK1 protein level was significantly decreased (*p* < 0.05), but those of Raf1 and ERK1/2 were unchanged (*p* > 0.05) (Fig. 6a), indicating that MEK1 is the target gene of miR-34a, which has also been demonstrated by others [23]. Moreover, protein levels of K-ras, p-Raf1, p-MEK1, and p-ERK1/2 were significantly down-regulated (*p* < 0.05) (Fig. 6a). We also detected MEK1, which is the most important molecule in the RAS/MAPK signal pathway in the anti-Thy1 mesangial proliferative glomerulonephritis rat model. Compared with 0 days, phospho-MEK1 at 7 days was significantly increased (*p* < *0.05*) (Fig. 6b). The above results suggested that Ras/MAPK signaling pathway plays a key role in the development of rat mesangial proliferative glomerulonephritis.

### Effects of siPDGFR-β on RMC cell cycle, cell cycle proteins, and the Ras/MAPK signaling pathway

**Fig. 7:**
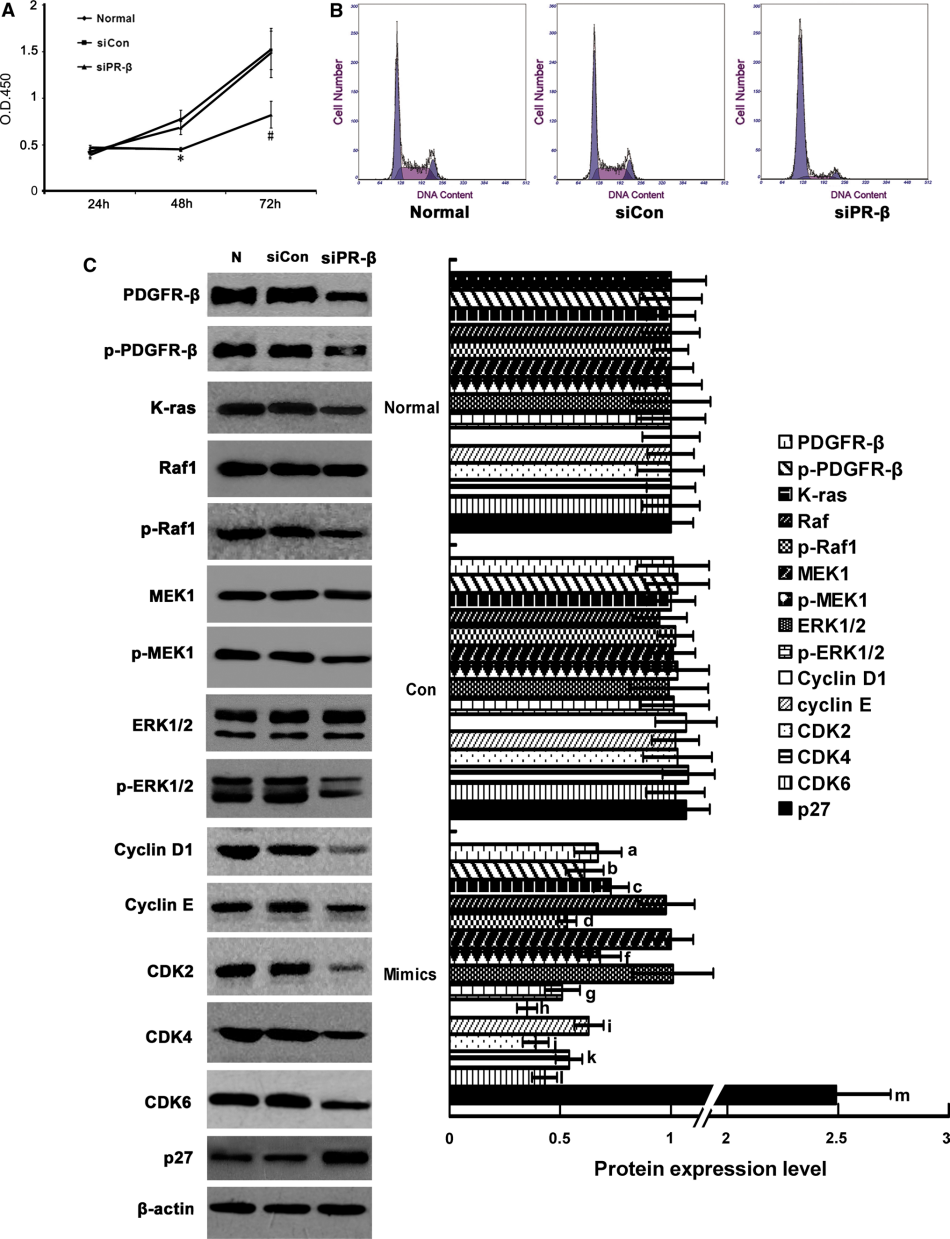
Effects of siPDGFR-β on proliferation, cell cycle, cell cycle proteins, and Ras/MAPK signaling pathway molecules in RMCs. **a** Proliferation activity of RMCs after transfection with siPDGFR-β or negative control was detected by the Cell Counting Kit-8. *p < 0.05 vs. normal group and siCON group at 48 h, *n* = 3; ^#^ *p* < 0.01 vs. normal group and siCON group at 72 h, *n* = 3. N: normal group; siCon: negative control group; siPR-β: siPDGFR-β. **b** FCM analysis of cell cycles of RMCs transfected with siPDGR-β or negative control. *p < 0.05 vs. normal group and siCon. group,* n* = 3. Normal: normal group; siCon: negative control group; siPR-β: siPDGFR-β. **c** Protein expression levels of PDGFR-β, p-PDGFR-β, cyclinD1, cyclinE, CDK2, CDK4, CDk6, p27^kip1^, K-ras, Raf-1, p-Raf-1, MEK1, p-MEK1, ERK1/2, p-ERK1/2 were detected with Western blot. Data are means of three separate experiments ± SD. ^a,b,c,d,e,f,g,h,i,j,k.l.m^ *p* < 0.05 vs. normal group and siCon group. Normal: normal group; siCon: negative control group; siPR-β: siPDGFR-β

To further determine whether miR-34a can directly target PDGFR-β, we evaluated the influences of siPDGFR-β on the cell cycle, cell cycle-related proteins, and Ras/MAPK signaling pathway molecules using an RNAi technique. After siPDGFR-β transfection, proliferation of RMC cells was suppressed (Fig. 7a; Table 6); and the G0/G1 phase was extended and the G2/M phase was reduced (Fig. 7b). Protein levels of cyclin D1, cyclin E, CDK2, CDK4, and CDK6 were significantly decreased after siPDGFR-β transfection (*p* < 0.05) (Fig. 7c). Levels of the Ras/MAPK signaling pathway proteins, K-ras, Raf1, MEK1, and ERK1/2, were determined. There were no changes in protein levels of total Raf1, MEK1, and ERK1/2 (*p* > 0.05) (Fig. 7d). However, the protein levels of K-ras, p-Raf1, p-MEK, and p-ERK1/2 were significantly decreased (*p* < 0.05) (Fig. 7d). These results indicate that siPDGFR-β directly suppressed PDGFR-β and then influenced the cell cycle through the Ras/MAPK signaling pathway. The actions of miR-34a and siPDGFR-β on both PDGFR-β and the cell cycle were similar.

**Table 6:** The percentage of mesangial cells in G0/G1, S, and G2/M phases

	G0–G1 (%)	S (%)	G2-M (%)
Normal	64.15 ± 2.84	22.68 ± 1.90	13.17 ± 1.88
siCon	62.71 ± 1.61	21.35 ± 2.31	15.59 ± 1.61
siPR-β	76.34 ± 2.32*	14.91 ± 1.57*	8.75 ± 0.84*

*Normal* normal group, *siCon* negative control group, *siPR-β* siPDGFR-β

* *p* < 0.05 vs. normal group and siCon group

### MiR-34a suppressed the proliferation of RMCs that were stimulated by 20 % FBS

**Fig. 8:**
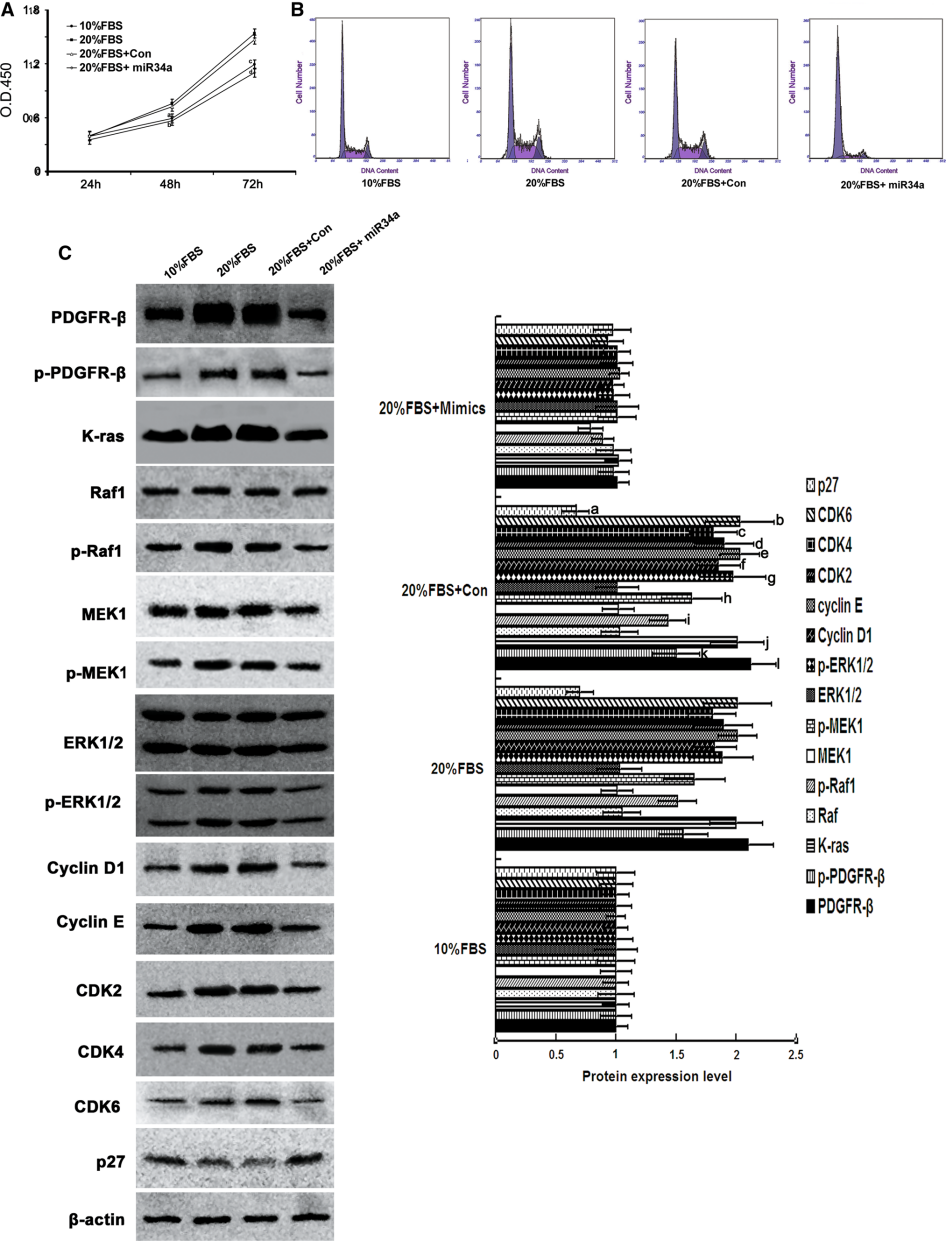
Effect of miR-34a on cell cycle, cell cycle proteins, and the Ras/MAPK signaling pathway molecules in the RMCs, which were stimulated to proliferate by 20 % FBS. **a** miR-34a mimics antagonize 20 % FBS-stimulate mesangial cell proliferation. RMCs cultured by 10 % FBS or 20 % FBS, and transfected with miR-34a mimics or negative control. The proliferation rate was detected by Cell Counting Kit-8. ^a,b^ *p* < 0.05 vs. 20 % FBS group and 20 % FBS + Con group at 48 h, *n* = 3; ^c,d^ *p* < 0.01 vs. 20 % FBS group and 20 % FBS + Con group at 72 h, *n* = 3. 10 % FBS: 10 % FBS group; 20 % FBS: 20 % FBS group; 20 % FBS + Con: 20 % FBS + negative control; 20 % FBS + miR-34a: 20 % FBS + miR-34a mimics. **b** FCM analysis of cell cycles of RMCs transfected with miR-34a mimics or negative control, *n* = 3. 10 % FBS: 10 % FBS group; 20 % FBS: 20 % FBS group; 20 % FBS + Con: 20 % FBS + negative control; 20 % FBS + miR-34a:20 % FBS + miR-34a mimics. **c** Protein expression levels of PDGFR-β, p-PDGFR-β, cycliyD1, cyclinE, CDK2, CDK4, CDk6, p27^kip1^, K-ras/Raf-1, p-Raf-1, MEK1, p-MEK1, ERK1/2, p-ERK1/2 were detected with Western blot. Data are means of three separate experiments ± SD. ^a,b,c,d,e,f,g,h,i,j,k,l^ *p* < 0.05 vs. 20 % FBS + mimics group. 10 % FBS: 10 % FBS group; 20 % FBS: 20 % FBS group; 20 % FBS + Con: 20 % FBS + negative control; 20 % FBS + miR-34a: 20 % FBS + miR-34a mimics

We have demonstrated that miR-34a can inhibit the proliferation of normal mesangial cell by directly inhibiting target gene PDGFR-β. Here, we stimulated mesangial cell proliferation with exogenous factors (20 % FBS), and then observed if miR-34a could antagonize this phenomenon. The results showed that, after 20 % FBS treatment, the proliferation rate of cultured mesangial cells was significantly higher than 10 % FBS-treated mesangial cells (*p* < 0.05) (Fig. 8a). After miR-34a mimics transfection, the proliferation rate of mesangial cells cultured with 20 % FBS was significantly lower than the control group (*p* < 0.05) and no difference with 10 % FBS culture group (*p* > 0.05) (Fig. 8a). Compared with the 10 % FBS group and 20 % FBS control group, the G0/G1 phase of RMCs cultured with 20 % FBS after miR-34a mimics transfection was extended, and the G2/M phase was reduced (Fig. 8b; Table 7). Compared with 20 % FBS control group, protein levels of cyclin D1, cyclin E, CDK2, CDK4, and CDK6 were significantly decreased in 20 % FBS-cultured RMC after miR-34a mimics transfection (*p* < 0.05) (Fig. 8c). Levels of the Ras/MAPK signaling pathway proteins, K-RAS, Raf1, MEK1, and ERK1/2, were determined. Compared with the 20 % FBS control group, K-RAS and total MEK1 protein levels of miR-34a mimics were decreased (*p* < 0.05), and the phosphorylated protein levels of Raf1, MEK1, ERK1/2 were significantly decreased (*p* < 0.05) (Fig. 8c). Compared with 20 % FBS-cultured group, which was transfected with miR-34a mimics, p27^kip1^ in the RMCs cultured with 20 % FBS was decreased (*p* < 0.05) (Fig. 8c). These results indicate that miR-34a directly suppressed the 20 % FBS-stimulated RMC proliferation.

**Table 7:** The percentage of mesangial cells in G0/G1, S, and G2/M phases

	G0–G1 (%)	S (%)	G2-M (%)
10 % FBS	58.556 ± 1.24	32.588 ± 1.38	8.856 ± 1.11
20 % FBS	47.436 ± 1.88	38.765 ± 2.10	13.798 ± 2.29
20 % FBS + Con	47.348 ± 2.57^a^	37.500 ± 2.31^b^	15.152 ± 1.53^c^
20 % FBS + miR34a	85..423 ± 3.23^d^	10.546 ± 1.80^e^	4.024 ± 2.20^f^

10 % FBS: 10 % FBS group; 20 % FBS: 20 % FBS group; 20 % FBS + Con: 20 % FBS + negative control; 20 % FBS + miR-34a: 20 % FBS + miR-34a mimics

^a,b,c^ *p* < 0.05 vs. 10 % FBS and 20 % FBS + miR-34a group, *n* = 3

^d,e,f^ *p* < 0.05 vs. 10 % FBS group, *n* = 3

## Discussion

MsPGN is divided into IgA and non-IgA nephropathy, and the main pathological changes associated with this condition are diffuse proliferation of GMCs and different degrees of ECM accumulation [1, 2, 24]. IgA nephropathy is the most common type of glomerulonephritis globally [25–27], and occurs mainly among Asians, especially the Japanese and Chinese [28–36]. A study of 13,519 Chinese renal biopsies found that primary MsPGN accounted for 70.88 % of all cases of glomerular diseases. IgA nephropathy comprised 45.26 % of the primary glomerular diseases, while non-IgA mesangial proliferative lesions comprised 25.62 % [37]. The risk factors associated with inflammatory reactions have not been determined. Mesangial cells are not only target cells of immune injury, but also proliferate under the influence of various stimulatory molecules, release inflammatory factors, and ECM components, and further promote kidney damage, ultimately leading to glomerular sclerosis and interstitial fibrosis.

miR-34 was first discovered in *C. elegans* in 2001 [14] and is associated with a variety of organ and tumor hyperplasias [15–20]. Therefore, this study aimed to investigate the role of miR-34a in proliferative glomerulonephritis. We first established an anti-Thy1 MsPGN rat model. In the anti-Thy1 glomerulonephritis rat model, we detected miR-34a expression in kidney tissues at various time points and found that miR-34a level gradually decreased as proliferation increased, then returned to normal levels when mesangial proliferation normalized. This indicates that miR-34a likely plays a suppressive role in RMC proliferation.

We found that the cell proliferation rate was lower in the miR-34a-transfected RMC group than in the control group, indicating that miR-34a inhibits the proliferation of RMCs. We then used flow cytometry to evaluate the influences of miR-34a on the cell cycle. In the miR-34a-transfected cells, G1/G0 was lengthened and the G2+M and S phases were shortened. Thus miR-34a can extend the G1 phase and inhibit cell proliferation. The above results are consistent with those reported for miR-34a in other tissues and tumors [16, 38, 39].

The most critical elements that positively regulate the G1 phase are cell cycle proteins (cyclins D1 and E). Cell cycle proteins and cell cycle protein kinases (CDK2, CDK4, and CDK6) are assembled into two types of complex: cyclin D1/CDK4/CDK6 and cyclin E/CDK2 [40, 41]. The cell cycle protein kinase inhibitor (CKI) p27^kip1^ inhibits cyclinD1/CDK4/CDK6 and cyclinE/CDK2 complexes, negatively regulating the cell cycle [42]. In the normal glomerulus, p27^kip1^ is highly expressed to maintain a steady state in the kidney and avoid over-proliferation [43]. The p27^kip1^ level dropped sharply and returned to baseline in the MsPGN model [43]. There was a similar change in PDGF-induced cell proliferation [44]. According to flow cytometry, miR-34a affects mainly the G0/G1 phase of the cell cycle; the main G0/G1 phase proteins cyclin D1, cyclin E, CDK2, CDK4, CDK6, and p27^kip1^ were evaluated in this study [1, 45, 46].

MiRanda, TargetScanS, and PicTar were integrated into MiRGen online biology prediction software (http://www.diana.pcbi.upenn.edu/miRGen.html), which is the bioinformatics software used most commonly to predict miRNA target genes. MiRGen prediction results suggested that PDGFR-β may be a direct target gene of miR-34a.

PDGFR is a receptor protein of PDGF. PDGF is classified into four subtypes, of which PDGF-B and PDGF-D are involved in the regulation of cell proliferation through PDGFR-β [46]. PDGF and PDGFR-β binding activates the Ras/MAPK signaling pathway as well as many downstream target genes, such as cyclins D1 and E [1], which control cell proliferation. The selective PDGFR tyrosine blocker STI157 can block mesangial cell proliferation [47].

We found that the PDGFR-β protein level was markedly lower in the miR-34a-transfected RMCs, while the mRNA level did not change. The results of the dual-luciferase assay indicated that PDGFR-β is a direct target gene of miR-34a. We assumed that cyclin E and CDK2 were target genes of miR-34a. The results of the dual-luciferase assay confirmed this hypothesis. MiR-34a not only regulates PDGFR-β expression but also acts directly in conjunction with cyclin E/CDK2 to regulate the cell cycle and influence proliferation. Other studies have confirmed that cyclin D1 [21], CDK4 [9], and CDK6 [21, 22] are target genes of miR-34a. In our study, there were no changes in the mRNA levels of cyclin D1, CDK2, or CDK6 while the proteins expression was significantly reduced after miR-34a transfection; however, the mRNA level of cyclin E was decreased in the miR-34a-transfected RMCs. Moreover, both p27^kip1^ protein and mRNA levels were increased in the miR-34a-transfected group, which is consistent with the effect on mesangial cell proliferation [44, 48].

To further define whether miR-34a regulates downstream target genes through the Ras/MAPK signaling pathway, we used miR-34a mimics to transfect RMCs. The total Raf1 and ERK1/2 protein level did not change, but those of K-RAS, p-Raf1, MEK1, p-MEK1, and p-ERK1/2 decreased. A previous study showed that the total Raf1, ERK1/2, or MEK1 protein level was unchanged when RAS/MAPK signaling pathways were activated. We hypothesize that the change in the total MEK1 protein level occurred because MEK1 is a target gene of miR-34a [23]. PDGFR-β activates the RAS/MAPK signal pathway molecules, K-RAS, p-Raf1, p-MEK1, and p-ERK1/2, thus affecting the G1 phase [49–52]. Therefore, miR-34a may directly suppress PDGFR-β and so regulate the expression of cell-cycle proteins (CDK and CKI) through the RAS/MAPK signaling pathway [1, 45].

To demonstrate that miR-34a modulates the cell cycle by directly inhibiting PDGFR-β, we used siPDGFR-β to inhibit the expression of PDGFR-β. The results showed that the changes in both cell proliferation and the cell cycle were identical to those in the miR-34a-transfected group after siPDGFR-β transfection. The cell cycle proteins also showed the same trend as in the miR-34a group. SiPDGFR-β and miR-34a exert similar influences on PDGFR-β and the cell cycle. Therefore, miR-34a can affect the G0/G1 phase through inhibition of PDGFR-β.

We have confirmed that miR-34a can directly inhibit the target gene PDGFR-β and in turn affect the normal RMC proliferation function by suppressing the G0/G1 phase. Here we use high concentrations of serum (20 % serum) to stimulate mesangial cell proliferation, and then use miR-34a to antagonize this phenomenon. The results are clear that high concentrations of serum could accelerate cell proliferation, however, the use of miR-34a together with a high concentration of serum in the cell, the cell proliferation rate return to normal levels for 10 % serum culture group. Thus, miR-34a can antagonize proliferation of the high concentration serum group.

**Fig. 9:**
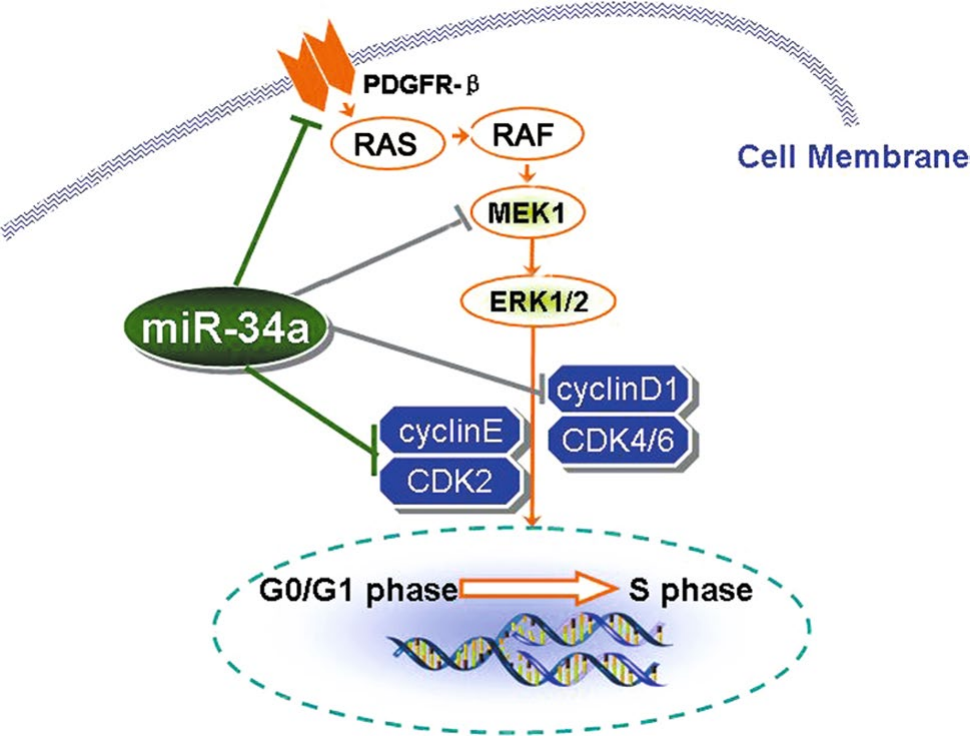
Proposed mechanisms by which miR-34a regulates mesangial cell proliferation

In conclusion, we found that miR-34a regulates mesangial cell proliferation by directly inhibiting expressions of PDGFR-β and factor (MEK1) in the RAS/MAPK signal pathway, and by influencing the G0/G1 phase. MiR-34a may also affect cell proliferation by directly suppressing the expression of cyclin E and CDK2 cell cycle proteins in the G0/G1 phase (Fig. 9).
